# The metabolic consequences of ‘yo-yo’ dieting are markedly influenced by genetic diversity

**DOI:** 10.1038/s41366-024-01542-2

**Published:** 2024-07-03

**Authors:** Senthil Thillainadesan, Aaron Lambert, Kristen C. Cooke, Jacqueline Stöckli, Belinda Yau, Stewart W. C. Masson, Anna Howell, Meg Potter, Oliver K. Fuller, Yi Lin Jiang, Melkam A. Kebede, Grant Morahan, David E. James, Søren Madsen, Samantha L. Hocking

**Affiliations:** 1 Charles Perkins Centre, University of Sydney, Camperdown, NSW 2006 Australia; 2 Faculty of Medicine and Health, University of Sydney, Camperdown, NSW 2006 Australia; 3 Department of Endocrinology, Royal Prince Alfred Hospital, Camperdown, NSW 2050 Australia; 4 School of Life and Environmental Sciences, University of Sydney, Camperdown, NSW 2006 Australia; 5 Australian Centre for Advancing Diabetes Innovations, Harry Perkins Institute of Medical Research, Nedlands, WA Australia

**Keywords:** Obesity, Diabetes

## Abstract

**Background:**

Weight loss can improve the metabolic complications of obesity. However, it is unclear whether insulin resistance persists despite weight loss and whether any protective benefits are preserved following weight regain (weight cycling). The impact of genetic background on weight cycling is undocumented. We aimed to investigate the effects of weight loss and weight cycling on metabolic outcomes and sought to clarify the role of genetics in this relationship.

**Method:**

Both C57BL/6 J and genetically heterogeneous Diversity Outbred Australia (DOz) mice were alternately fed high fat Western-style diet (WD) and a chow diet at 8-week intervals. Metabolic measures including body composition, glucose tolerance, pancreatic beta cell activity, liver lipid levels and adipose tissue insulin sensitivity were determined.

**Results:**

After diet switch from WD (8-week) to chow (8-week), C57BL/6 J mice displayed a rapid normalisation of body weight, adiposity, hyperinsulinemia, liver lipid levels and glucose uptake into adipose tissue comparable to chow-fed controls. In response to the same dietary intervention, genetically diverse DOz mice conversely maintained significantly higher fat mass and insulin levels compared to chow-fed controls and exhibited much more profound interindividual variability than C57BL/6 J mice. Weight cycled (WC) animals were re-exposed to WD (8-week) and compared to age-matched controls fed 8-week WD for the first time (LOb). In C57BL/6 J but not DOz mice, WC animals had significantly higher blood insulin levels than LOb controls. All WC animals exhibited significantly greater beta cell activity than LOb controls despite similar fat mass, glucose tolerance, liver lipid levels and insulin-stimulated glucose uptake in adipose tissue.

**Conclusion:**

Following weight loss, metabolic outcomes return to baseline in C57BL/6 J mice with obesity. However, genetic diversity significantly impacts this response. A period of weight loss does not provide lasting benefits after weight regain, and weight cycling is detrimental and associated with hyperinsulinemia and elevated basal insulin secretion.

## Introduction

Obesity is associated with insulin resistance, Type 2 diabetes and other metabolic complications. Weight loss can lead to improvements in many of these metabolic complications and even remission of Type 2 diabetes [1, 2]. However, following weight loss, weight maintenance is challenging and weight regain is common. The pattern of weight loss and regain is often termed “yo-yo dieting” or weight cycling. Of concern, a number of observational human studies suggest that weight cycling is associated with poor cardiometabolic outcomes and increased risk of Type 2 diabetes [3, 4].

Mouse models of weight cycling, which involve alternating between high fat and chow diets, have demonstrated the potential metabolic harm of weight cycling with greater adiposity and glucose intolerance after weight regain [5–9]. However, these studies have predominantly been performed in C57BL/6 J mice, which demonstrate a robust and rapid normalisation of weight, body composition and metabolic parameters following diet-induced weight loss. This is in stark contrast to the significant variability seen in human studies [10–12] and raises the possibility that the results of weight loss and weight cycling studies in C57BL/6 J mice may not be generalisable to other genetic backgrounds. Studies in humans have found significant interindividual variability in weight loss response following dietary or lifestyle interventions. Genetics, among other factors, is thought to be one of the main drivers of this variability [13–15]. Specifically, emerging evidence indicates that an individual’s weight loss trajectory throughout their lifespan has a significant genetic contribution [16] and recent genome-wide association studies have identified numerous loci associated with the degree of weight loss after dietary intervention [15]. Overall, the contribution of genetics to weight loss remains poorly understood, and no study to date has examined the effects of weight cycling on animals with genetically diverse backgrounds.

The Diversity Outbred (DO) model is a population of mice generated through the outbreeding of 8 founder strains, which comprise both classic and wild-derived inbred strains. The DO mouse population contains more genetic diversity than any other such population and parallels the diversity seen in human populations [17]. Moreover, DO mice display marked variability in glucose levels, insulin measures and ex-vivo islet insulin secretion following feeding with a high fat high sugar Western-style diet (WD) [18]. This population is therefore a valuable resource for studying how genetics influence the response to weight loss interventions and for examining whether the findings from studies on inbred C57BL/6 J mice can be generalised to diverse genetic backgrounds.

Insulin resistance and impaired insulin action in adipose tissue is a key defect in the development of the metabolic syndrome and has been found to precede other metabolic abnormalities seen with obesity, often developing rapidly in mouse models following exposure to WD [19–22]. Thus, we were interested in assessing whether the defects in insulin action in these tissues persist following weight reduction and whether weight cycling leads to further impairment in insulin sensitivity. We hypothesised that having a history of obesity (having been overweight but subsequently losing weight) may result in residual defects in insulin action, particularly in adipose tissue, and ultimately may contribute to worse metabolic outcomes upon weight regain.

In the present study, we sought to investigate the detrimental effects of weight cycling by comprehensively profiling metabolic outcomes following both weight loss and regain in weight cycled (WC) animals. Importantly, we aimed to gauge the impact of genetic differences on this relationship by determining whether the metabolic response to weight cycling seen in C57BL/6 J mice was paralleled in genetically diverse Diversity Outbred Australia (DOz) mice. We found that C57BL/6 J and DOz mice had distinct responses to the same dietary weight loss intervention where C57BL/6 J but not DOz exhibited a robust reduction in fat mass and normalisation of metabolic outcomes. Interestingly, WC C57BL/6 J and DOz mice both displayed basal hypersecretion of insulin following re-exposure to WD.

## Materials and Methods

### Animals

All animal experiments were performed in accordance with NHMRC (Australia) guidelines and were approved by the University of Sydney animal ethics committee (Protocol no 2020/1790). Animals were assigned to cages of 2–5 animals/cage and were housed at 23 °C on a 12 h light-dark/cycle and given *ad libitum* access to diet and water. Mice were acclimatised for 1 week prior to experimentation. Male C57BL/6 J mice (12 week old) were purchased from the Animal Resource Centre (Perth, Australia).

#### Diversity Outbred Australia (DOz) mice

All DOz mice used in the study were generated at the University of Sydney [23]. DOz mice were outbred from 29 breeding pairs of mice from 55 independent partially inbred lines of the Collaborative Cross (CC) mice from the Geniad colony in Western Australia (Collaborative Cross Consortium 2012). The CC mice were derived from 8 inbred founder strains, which display greater genetic variation than existing recombinant inbred strains and other mouse genetic reference populations. The strains include classic inbred strains A/J, C57BL/6 J, 129S1/SvImJ, NOD/ShiLtJ, and NZO/HlLtJ and 3 wild derived inbred strains CAST/EiJ, PWK/PhJ, and WSB/EiJ. Each CC line used for the generation of DOz was produced by an 8-way cross using the founder strains in a randomised order [17]. The DOz colony was generated and maintained using a breeding strategy that avoids mating siblings or first cousins, using one male and female pup from each litter in a manner analogous to that used to generate the Jackson Lab Diversity Outbred mice [17]. The number of breeding pairs was increased to 46 at the time the colony was established at the University of Sydney [23]. Male DOz mice from generations 36 and 37 of outbreeding were used in this study and were weaned onto a chow diet.

### Experimental Design

#### C57BL/6 J Study Design

To investigate whether defects in peripheral insulin action following diet-induced obesity persist despite weight reduction, C57BL/6 J mice (12 weeks old) were randomised via a random number generator into 3 groups (*n* = 6–8/group): (1) a control group fed a standard rodent chow diet for 16 weeks; (2) a weight loss group (WL) fed a WD for 8 weeks to induce obesity and then switched to a chow diet for 8 weeks to induce weight loss; (3) an always having Obesity (AOb) group fed WD for 16 weeks. Animals were euthanised at 16 weeks by cervical dislocation for tissue collection and glucose uptake assays.

To study the effects of weight cycling on metabolic health, C57BL/6 J mice (12 week old) were randomised via a random number generator into four groups (*n* = 12–14/group): (1) a control group fed a standard rodent chow diet for the entire 24-week study duration; (2) a WC group exposed to a WD for 8 weeks to induce obesity, then fed a chow diet for 8 weeks to induce weight loss and then re-exposed to a WD for 8 weeks; (3) a later-onset obesity (LOb) group which were fed a chow diet for 16 weeks and challenged with WD for the final 8 weeks of the study; (4) an AOb group which were fed WD for the entire 24-week study duration. Mice were euthanised by cervical dislocation for tissue collection, glucose uptake assays and islet insulin secretion assays at 24 week.

#### DOz Mice Study Design

DOz mice (14 week old) were randomised via a random number generator into two groups (*n* = 30–32/group): (1) A WC group alternated between 8 weeks of western diet (WD), 8 weeks of chow diet and 8 weeks of WD; (2) A control LOb group fed chow diet for 16 weeks followed by WD for the final 8 weeks. DOz mice exhibit much greater phenotypic variability than C57BL/6 J mice, warranting a larger sample size. Based on previous work in DOz mice and the fact that genetic mapping was not performed, *n* = 30–32 was deemed sufficient for the present study [17]. Mice were euthanised by cervical dislocation at the end of the 24 weeks study for tissue collection.

#### DOz mice Energy Intake Study Design

At 12 weeks of age, male DOz mice were randomised via a random number generator into two groups (*n* = 19–20/group): (1) a WC group alternated between 2 weeks of WD, 2 weeks of chow and 2 weeks of WD; (2) a control LOb group fed chow for 4 weeks followed by WD for the final 2 weeks. Mice were pair-housed with a cage divider, enabling individual energy intake measurements to be collected. Daily energy intake was determined by measuring the weight of the input food minus the weight of the leftover food after 24 h, multiplied by caloric density. The daily energy intake was measured 3 days per week for each of the 2nd, 4th, and 6th week.

#### Mouse Diets

The control chow diet contained 12% calories from fat, 65% calories from carbohydrates, and 23% calories from protein (‘Irradiated Rat and Mouse Diet’, Specialty Feeds, Glen Forest, WA, Australia). The WD was made in house, containing 45% calories from fat, 36% calories from carbohydrates and 19% calories from protein [24].

### Experiments

#### Metabolic Data Collection

Body weight was measured weekly. Body composition was assessed using nuclear magnetic resonance, EchoMRI (EchoMRI Corporation Pte Ltd, Singapore) at baseline and every 2 weeks from the 8 week time point onwards to monitor lean and fat mass. An oral glucose tolerance test (GTT) was performed at the 6, 14 and 22 week time points. Mice were fasted for 6 h and orally gavaged with 20% glucose in water at 2 mg/kg lean mass. Blood glucose levels were monitored at baseline, 15, 30, 45, 60 and 90 min using a glucose monitor (Accu-Chek, Roche Diabetes Care, NSW, Australia). Blood (5 μL) was also collected at baseline and 15 min directly into an Insulin Mouse Ultra Sensitive ELISA kit (Cat# 90080; Crystal Chem USA, Elk Grove Village, Illinois, USA) to determine insulin levels.

Blood was collected at the 24 week timepoint in EDTA-coated tubes from fasted animals via saphenous vein bleeds after a 6 h fast and centrifuged at 2000 g for 15 min to isolate plasma.

#### Adipose Tissue Glucose Uptake

Adipose tissue glucose uptake was measured using previously described methods [24]. epididymal white adipose tissue (eWAT) explants were finely minced and were incubated in 25 mM glucose DMEM (Gibco #12800-017), with 25 mM Hepes and 2% BSA at pH 7.4 and at 37 °C for 2 h. eWAT explants were then incubated in KRP containing 25 mM Hepes and 2% BSA for 20 min with either 0 or 10 nM insulin and 50 μM 2-deoxyglucose (DG), 1 μCi [^3^H]-2-DG and 0.14 Ci [^14^C]-mannitol for the final 5 min in technical duplicates. Glucose uptake was stopped with 3 washes of ice-cold PBS and followed by lysis in 100 mM sodium hydroxide. The 2-[^3^H]DG tracer content was quantified by liquid scintillation spectroscopy, corrected for extracellular [^14^C]mannitol counts and normalised to protein content.

#### Islet Ex-vivo Insulin Secretion Assay

Pancreatic islet isolation and insulin secretion assays were performed according to previously described methods [25]. Pancreata were inflated by injection of the common bile duct with 0.25 mg/mL Liberase (Roche Applied Science) in Hanks’ buffered saline solution (Life Technologies) with 20 mM HEPES. Pancreata were digested for 13 min at 37 °C, washed, filtered through 1000 mm mesh and subjected to Histopaque 1119 and 1077 (Life Technologies) gradient. Islets were handpicked under a microscope. Isolated islets were pre-incubated at 37 °C in Krebs–Ringer bicarbonate HEPES containing 2.8 mM glucose for 2 h prior to incubation in either low (2.8 mM) or high (16.8 mM) glucose for 1 h. Insulin secretion was measured from static (1 h) incubations and expressed as ng/mL insulin secreted in this time per islet. An assay was performed using 5 islets per mouse.

#### Plasma Assays

Plasma levels of non-esterified fatty acid (NEFA) levels (NEFA C kit, WAKO, Richmond, VA, USA) were quantified using enzymatic colorimetric assays as per manufacturer instructions. Plasma FABP4 levels were quantified using an ELISA kit (Circulex CY-8077).

#### Liver Lipid Assays

Frozen liver tissue was powdered and lipids were extracted using 2:1 chloroform:methanol solution and dried down using nitrogen. Samples were resuspended in isopropanol and triglyceride and cholesterol content were measured using colorimetric enzymatic assays (ThermoFischer Scientific 981786 and TR13421, respectively).

### Statistical Analysis

Statistical analysis was performed using R 4.0.4. ANOVA was used for multiple group comparisons, and Student’s *t*-test or Mann–Whitney *U*-Test for comparisons between two groups.

### Equation for HOMA-B

The homeostatic assessment of beta cell function (HOMA-B) was calculated as below [26].

HOMA-B = 20 * fasting insulin (mU/mL)/ (fasting glucose (mmol/L) - 3.5)

## Results

### *Western-to-chow diet switch results in normalisation of metabolic parameters in C57BL/6J mice*

**Fig. 1:**
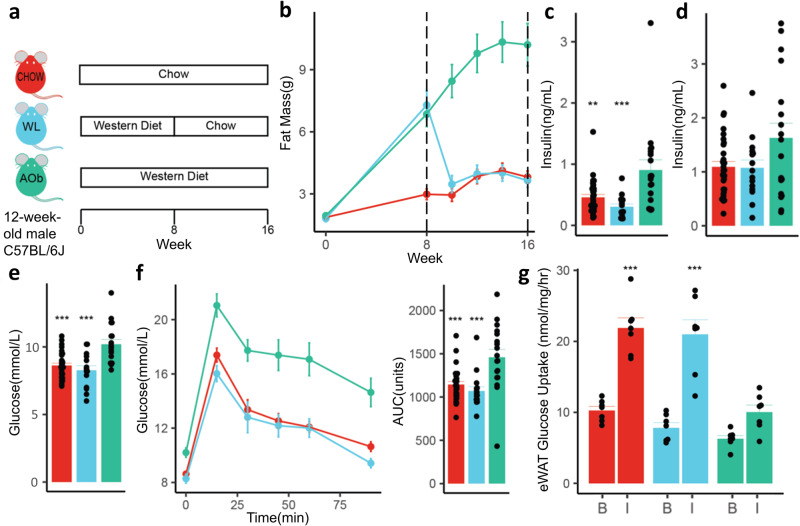
Effect of weight loss on metabolic outcomes in C57BL/6 J mice. **a** Weight loss study design, (**b**–**g**) metabolic outcomes at 16 wk time point including (**b**) fat mass, (**c**) fasting insulin, (**d**) insulin at 15 min time point during glucose tolerance test (GTT) (**e**) fasting glucose, (**f**) GTT results and area under the GTT curve, (**g**) ex-vivo 2-deoxyglucose (2DG) uptake basal (0 nM) and insulin stimulated (10 nM) into adipose tissue. Error bars are SEM. *p < 0.05, **p < 0.01, ***p < 0.001 vs AOb (*n* = 6–16/group). All statistical analysis was conducted using ANOVA. WL: Weight loss, AOb: Always having Obesity.

To investigate whether a history of obesity leads to any persistent metabolic defects following a reduction in weight, we fed male C57BL/6 J mice a Western diet (WD) for 8 weeks to induce obesity followed by a chow diet for 8 weeks. The weight loss group (WL) was compared to chow fed controls as well as always having obesity animals (AOb), which were fed WD for the entire 16 weeks (Fig. 1a). As expected, WD feeding resulted in increased weight (Supplementary Fig. 1b), fat mass (Fig. 1b), hyperinsulinemia (Fig. 1c, d) and glucose intolerance (Fig. 1e, f). The WL group exhibited a rapid reduction in fat mass reaching comparable adiposity to chow fed controls within 4 weeks of switching diets (Fig. 1b). The glucose intolerance and hyperinsulinemia arising from WD feeding also normalised following the switch to chow (Fig. 1c–f). Next, we looked for residual effects on tissue insulin responsiveness by measuring insulin-stimulated glucose uptake in adipose tissue explants. As expected, the AOb mice developed peripheral insulin resistance with reduced insulin-stimulated glucose uptake into adipose tissue (Fig. 1g), compared to both the WL and chow control groups. Importantly, there was no significant difference between the WL animals and the chow fed controls, indicating that weight loss leads to the reversal of insulin resistance in adipose tissue (Fig. 1g). The WL and chow-fed groups additionally had comparable liver lipid content, which were both significantly lower than the liver lipids in the AOb group (Supplementary Fig. 1c, d).

### *Weight cycling results in the recurrence of metabolic consequences in C57BL/6J mice*

**Fig. 2:**
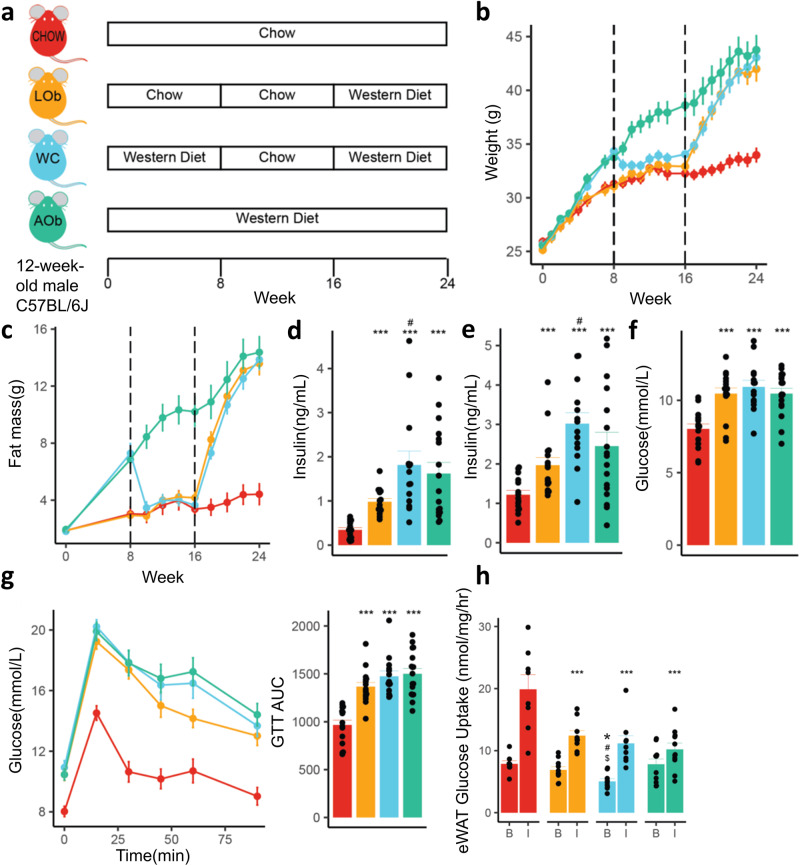
Effect of weight cycling on metabolic outcomes in C57BL/6 J mice. **a** Weight cycling experimental design for 24-week study duration, **b** group means for body weight over 24 weeks, (**c**) group means for fat mass over 24 weeks, (**d**–**h**) metabolic outcomes at 24 week time point including (**d**) fasting insulin, (**e**) insulin at 15 min during glucose tolerance test (GTT), (**f**) fasting glucose, (**g**) GTT results and area under the GTT curve, (**h**) ex-vivo glucose uptake basal (0 nM) and insulin stimulated (10 nM) into adipose tissue. Error bars are SEM. *p < 0.05, **p < 0.01, ***p < 0.001 vs chow, #*p* < 0.05 vs LOb, $*p* < 0.05 vs AOb (*n* = 6–16/group). All statistical analysis was conducted using ANOVA. LOb Later onset obesity, WC Weight cycling, AOb Always having obesity.

To explore whether a history of obesity predisposes animals to worse metabolic outcomes when re-exposed to an obesogenic environment, animals were fed WD for 8 weeks to induce obesity, then chow diet for 8 weeks to normalise weight, and lastly a second exposure to WD for another 8 weeks resulting in weight regain (weight cycled (WC) animals). To control for age, another group of age-matched chow-fed animals with no previous history of obesity were fed WD for 8 weeks. We term this group Later-onset Obesity animals (LOb) (Fig. 2a). The WC and LOb animals gained weight at a comparable rapid rate during the final 8 weeks of WD feeding reaching a similar body weight and fat mass to AOb animals (Fig. 2b, c). The WC, LOb, and AOb groups all developed hyperinsulinemia (Fig. 2d, e) and glucose intolerance (Fig. 2f, g) compared to the chow group. Interestingly, the WC animals had significantly higher levels of insulin compared to the LOb controls both at fasting and at 15 min during the glucose tolerance test (Fig. 2d, e), suggesting that a history of obesity may influence peripheral insulin sensitivity and/or beta cell function.

Next, ex-vivo glucose uptake assays into adipose tissue were performed to determine whether differences in peripheral insulin resistance were responsible for the hyperinsulinemia observed with weight cycling. Contrary to this prediction, insulin-stimulated glucose uptake into adipose tissue did not significantly differ between the WC, AOb and LOb animals, although basal glucose uptake was significantly lower in the WC group compared to all others (Fig. 2h). Similarly, hepatic triglyceride and cholesterol levels were found not to significantly differ between WC, LOb and AOb animals (Supplementary Fig. 2b, c). Overall, these results suggest that hyperinsulinemia in WC animals is not driven by adipose insulin resistance or intrahepatic lipids.

### *Western-to-chow diet switch does not normalise adiposity and hyperinsulinemia in DOz mice*

**Fig. 3:**
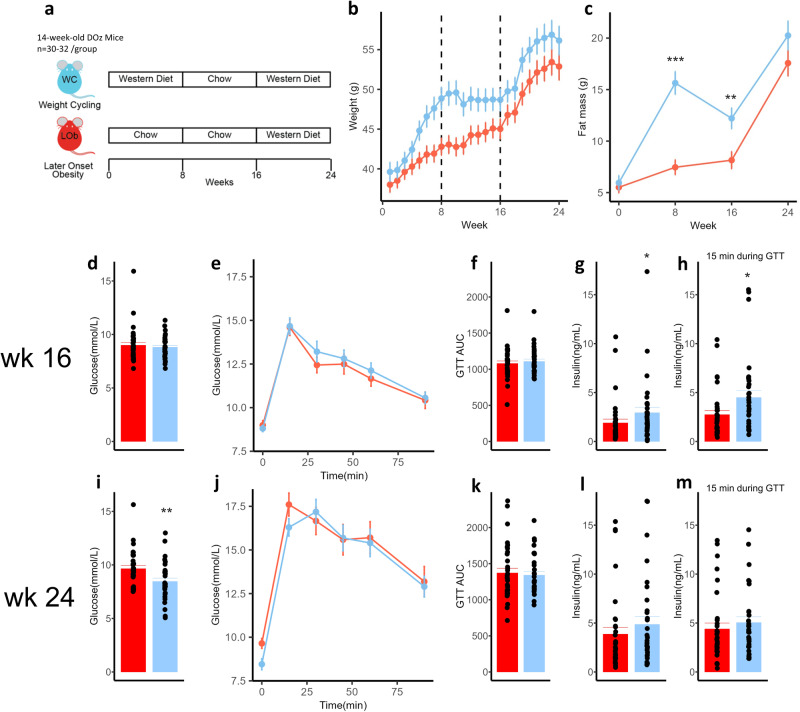
Effect of weight cycling on metabolic outcomes in DOz mice. **a** Experimental design for DOz mice study. Group means over 24 weeks for (**b**) body weight, (**c**) fat mass. Group means at 16 weeks time point for (**d**) fasting glucose, (**e**) GTT results (**f**) area under the GTT curve, (**g**) fasting insulin, (**h**) insulin at 15 min during GTT. Group means at 24 weeks time point for (**i**) fasting glucose, (**j**) GTT results, (**k**) area under the GTT curve, (**l**) fasting insulin, (**m**) insulin at 15 min during GTT. Error bars are SEM. Statistical analysis was conducted using two-sample *T*-tests for (**c**, **d**, **f**, **i**, **k**, **l**) or Mann–Whitney *U*-test where groups had unequal variances or outliers (**g**, **h**). *p < 0.05, **p < 0.01, ***p < 0.001 (*n* = 30–32/group). LOb Later onset obesity, WC Weight Cycling.

C57BL/6 J mice have a predisposition to developing hyperinsulinemia with WD feeding and do not capture the genetic diversity which influences the response to weight loss interventions in human studies [13, 27]. Other strains of mice have tremendous variation in insulin secretion and previous studies in Diversity Outbred mice have demonstrated over 1000-fold range in insulin secretion on a western diet [18]. To explore how genetic diversity influences the outcomes following weight cycling, we used Diversity Outbred Australia (DOz) mice. Similarly as was done for the C57BL/6 J mice, a WC group of DOz mice were alternated between Western and chow diets and compared to a group of age-matched LOb controls (Fig. 3a).

As expected, WD feeding in the first 8 weeks led to increased weight and fat mass in the WC group compared to the LOb control group (Fig. 3b, c). When the WC group was switched back to a chow diet, body weight and fat mass decreased, and fasting blood glucose and glucose tolerance returned to baseline (Fig. 3b–f). However, interestingly, the average fat mass of the WC group remained significantly higher by 44% than that of the LOb control group (Fig. 3c). The final 8 wks of WD feeding resulted in increased mean body and fat mass with similar rates, independent of weight cycling (Fig. 3b, c). Surprisingly, the WC group had significantly lower glucose levels than the LOb controls at the 24 week timepoint by approximately 1 mM, yet comparable glucose tolerance (Fig. 3i–k). Fasting insulin levels and insulin at 15-min during GTT remained significantly elevated in the WC animals compared to LOb controls at the 16-weeks time point before converging at the 24-week timepoint (Fig. 3g, h, l, m). Overall, in contrast to C57BL/6 J mice, these findings demonstrate that genetically diverse WC mice do not exhibit normalisation of fat mass and insulin levels upon switching from WD to chow. Also in contrast to C57BL/6 J mice, WC DOz mice do not exhibit significantly greater hyperinsulinemia than LOb controls at the 24-week timepoint.

### *The dietary intervention response of DOz mice is more variable than and distinct from C57BL/6J mice*

**Fig. 4:**
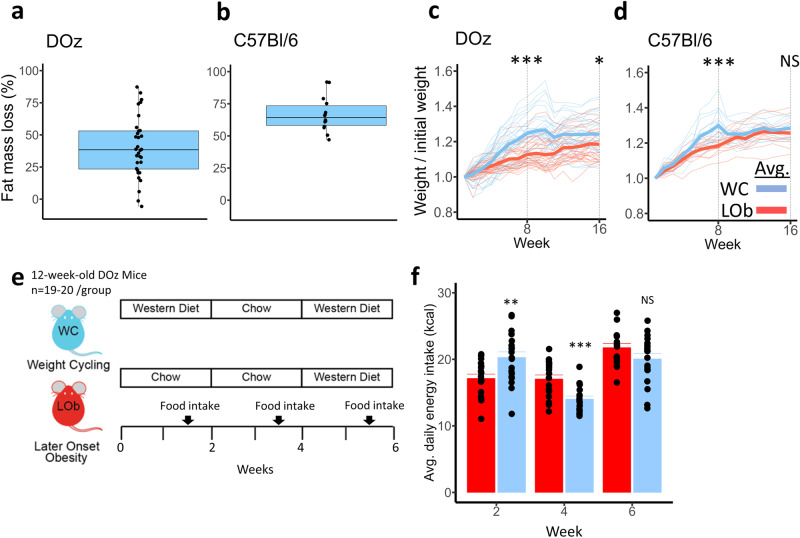
Weight Cycling responses and energy intake in DOz and C57BL/6 J mice. Fat mass loss (%) following switch from WD to chow for (**a**) DOz mice and (**b**) C57BL/6 J mice. Fat mass loss (%) = -(fat mass week 16 - fat mass week 8) / (fat mass week 8 - fat mass week 0). Adjusted weight over 24 weeks in (**c**) DOz mice and (**d**) C57BL/6 J mice. Adjusted weight = weight / initial weight (**g**). Opaque lines are group averages per week, translucent lines are individual values per week. **e** Experimental design for the DOz mice energy intake study. **f** Group means over 6 weeks for Average daily energy intake (kcal). Energy intake was measured for 3 consecutive days every 2nd week and averaged to gauge the average daily energy intake per mouse for weeks 2, 4, and 6. Error bars are SEM. NS not significant, *p < 0.05, **p < 0.01, ***p < 0.001 (*n* = 14–16 / group for C57BL/6 J study, *n* = 30–32/group for DOz study, *n* = 19–20 / group for DOz energy intake study). All statistical analysis was conducted using two-sample *T*-tests. LOb Later onset obesity, WC Weight Cycling.

Metabolic outcomes returned to baseline following a switch from WD to chow in C57BL/6 J but not in DOz mice. We wanted to explore these inconsistencies further and speculated that DOz mice were more resistant to improvements in body composition following the diet change. Figure 4a, b display the change in fat mass following the switch from WD to chow as a percentage of fat gain in the initial 8 weeks of WD feeding. As predicted the genetically diverse DOz mice exhibited greater heterogeneity and were on average much less responsive to the weight loss intervention. Only ~30% of animals (10/32) lost more than 50% of the fat gained in the first 8 weeks and 2 animals even increased fat mass despite switching to chow. In contrast, C57BL/6 J mice demonstrated a much more sensitive and homogeneous response to the weight loss intervention with all but 1 of the animals losing over 50% of their initially gained fat mass.

The weight of DOz mice at the start of the study was 46% higher on average than that of the C57BL/6 J mice and 4 times more variable when considering the standard deviation (sd_DOz_ = 6.26 g, sd_C57BL/6J_ = 1.55 g). We were interested in the variability in the weight response to diet switching, not the variability in the weight itself, and hence we accounted for the differences in starting weight by dividing each animal’s weight throughout the 16 wks by its starting weight. As a group, the adjusted weights of WC DOz mice separated from the age-matched LOb mice following the initial WD feeding and remained elevated thereafter, whereas that of WC C57BL/6 J mice was only temporarily elevated and quickly converged with LOb mice following the switch to chow (Fig. 4c, d). This difference in adjusted weights between WC and LOb groups remained significantly elevated at 16 weeks in DOz but not C57BL/6 J mice. The interindividual variability in adjusted weight was also greater for DOz compared to C57BL/6 J mice. Overall, the DOz mice’s fat mass and weight phenotypes were not only more variable but also strikingly distinct from C57BL/6 J mice.

DOz mice appear to be more resistant to improvements in body composition following the Western-to-chow diet switch compared to C57BL/6 J mice. One possibility is that after switching diets, DOz mice compensate by overeating the chow diet. In C57BL/6 J mice, in the 2nd week following a WD to chow switch, energy intake decreases by ~48% and is ~25% lower than that of chow-fed controls [28]. Energy intake in WC DOz mice, however, is undocumented. To scrutinise whether WC DOz mice overeat chow diet, we conducted a 6 weeks follow-up study on 40 pair-housed but separated mice, enabling energy intake per mouse to be measured (Fig. 4e). Energy intake was measured every second week, permitting the mice a week to acclimatise following diet changes. As expected, the WC group had significantly greater average daily energy intake during the initial WD feeding period when compared to the LOb controls (Fig. 4f). When switched to chow, the energy intake of the WC group decreased by 31% and was significantly lower than the LOb controls by 18%. This decrease is lower than that of C57BL/6 J mice, but overall, largely comparable [28, 29]. These data indicate that the WC DOz mice did not overeat during the weight loss phase, and thus this cannot explain their resistance to improvements in body composition.

### Weight cycled animals have increased ex-vivo islet insulin secretion and HOMA-B scores compared to AOb, LOb and chow groups

**Fig. 5:**
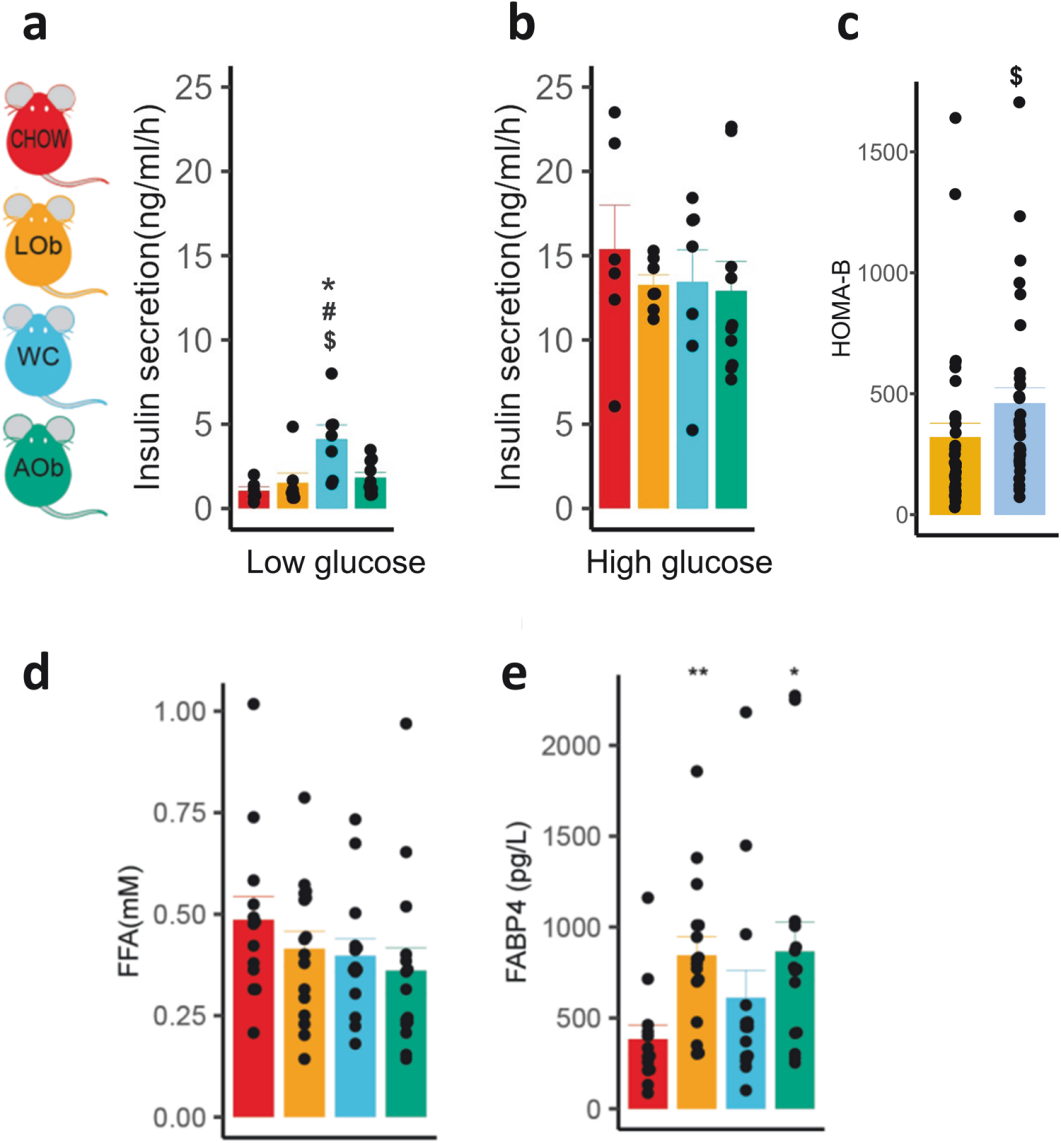
Effect of weight cycling on insulin secretion in C57BL/6 J and DOz mice. Islet insulin secretion in chow, LOb, WC and AOb C57BL/6 J mice following (**a**) low dose (2.8 mM) or (**b**) high dose (16.7 mM) glucose, (**c**) Homeostasis Model Assessment of beta cell function (HOMA-B) in LOb (orange) and WC (blue) DOz mice, (**d**) plasma FFA in chow, LOb, WC and AOb C57BL/6 J mice after a 6 h fast, (**e**) plasma FABP4 levels in chow, LOb, WC and AOb C57BL/6 J mice. All measurements taken at 24 week time point. *p < 0.05, **p < 0.01 vs chow, #*p* < 0.05 vs AOb, $*p* < 0.05 vs LOb (*n* = 6–32/group) Statistical analysis was conducted using ANOVA (**a**, **b**, **d**, **e**) or Mann–Whitney *U*-test (**c**). LOb Later onset obesity, WC Weight cycling, AOb Always having Obesity.

Our findings in C57BL/6 J mice indicated that hyperinsulinemia in WC animals was not driven by adipose insulin resistance or intrahepatic lipids. Therefore, we speculated that pancreatic beta cell dysfunction might instead be the root cause of hyperinsulinemia. Beta cells from WC animals demonstrated significantly greater insulin secretion in response to low glucose compared to all other groups (Fig. 5a), whereas no differences in insulin secretion were detected during high glucose stimulus (Fig. 5b). Interestingly, although insulin secretion was not directly measured in DOz mice, the WC DOz mice did exhibit significantly higher HOMA-B at the 24 week time point when compared to LOb controls indicating higher basal insulin secretion, consistent with the C57BL/6 J mice (Fig. 5c).

Free fatty acids (FFAs) are known to potentiate islet insulin secretion, and chronic exposure is thought to increase basal insulin secretion but reduce the response to glucose stimuli [30]. We speculated that reprogramming of islets due to chronic elevation of FFA levels may be the mechanism for increased basal insulin secretion in WC animals. However, fasting plasma FFA levels were not different between WC and LOb C57BL/6 J mice (Fig. 5D). Next, we explored whether the adipokine FABP4 may instead be responsible. FABP4 has been identified as a regulator of islet function in obesity [31, 32], where higher levels of FABP4 are documented in individuals with obesity and FABP4 was shown to elevate insulin secretion. LOb and AOb animals exhibited elevated FABP4 levels compared to chow-fed controls, whereas, contrary to our prediction, WC animals did not (Fig. 5e). Overall, FFAs and FABP4 are unlikely drivers of the elevated basal insulin secretion observed in WC animals.

## Discussion

Using a dual approach in C57BL/6 J and DOz mice, the present study investigated the effects of weight loss and weight cycling on metabolic outcomes and sought to clarify the role of genetics in this relationship. Our main findings were twofold: Firstly, reduction in adiposity and improvements in metabolic outcomes following a dietary weight loss intervention were found to greatly depend on genetics. Secondly, WC animals were found to be predisposed to hyperinsulinemia and elevated basal insulin secretion following re-exposure to WD.

Insulin resistance is considered to be an early defect in the development of diabetes and metabolic diseases, where impairment in insulin action predates other metabolic abnormalities [19]. Furthermore, inflammation and altered immune cell subpopulations – secondary to obesity – have been documented to persist in adipose tissue following weight loss [33]. Accordingly, we hypothesised the same may be true for insulin action, where defects may persist in adipose tissue upon weight loss, despite improvements in other metabolic outcomes. Our results conversely indicated that insulin responsiveness in adipose tissue returned to baseline following weight loss in C57BL/6 J mice. Moreover, although hepatic insulin resistance was not measured, we did observe a significant reduction in hepatic triglyceride content (a surrogate measure for hepatic insulin resistance [34]) following weight loss. Overall, our C57BL/6 J mice results showed that following dietary intervention weight, adiposity, peripheral insulin resistance, and ectopic lipid deposition all completely reversed to levels of chow-fed animals.

Human studies have contrastingly found that weight, ectopic lipid deposition and adipose tissue inflammation are reduced but remain elevated following a weight loss intervention in individuals with obesity compared to healthy controls [35]. Similarly, the rapid normalisation of weight, adiposity and hyperinsulinemia seen in C57BL/6 J mice was not consistent with DOz mice following the same Western-to-chow diet switch. Based on our work in the DOz population, we suggest that the response of C57BL/6 J mice is idiosyncratic and is evidently not generalisable to diverse mouse strains. In-line with this conclusion, strain-specific metabolic responses to obesogenic environments are widely documented [24, 36]. For example, unlike C57BL/6 J mice, WD-fed A/J, BALB and CAST are often protected against hallmarks of WD-induced metabolic disease. Hence, it is problematic that almost all animal studies gauging the metabolic consequences of weight cycling are conducted in C57BL/6 mice [8].

Considerable interindividual variability to any weight loss intervention is widely documented in humans [13]. Here, we find clear parallels in our DOz population. Differences in adherence to lifestyle changes, microbiome effects and genetics have been raised as potential explanations for recorded variability in weight loss trials [15, 37]. Human studies however, often fail to control for environmental factors, which may confound the weight loss response making it difficult to delineate the interaction between genetics and the environment. Here, we address this limitation and take advantage of the DOz population where confounding environmental factors can be largely controlled for. In doing so, the observed differences in metabolic outcomes, following a weight loss intervention, can be attributed to genetic variation.

Follow-up studies of individuals who underwent weight loss through lifestyle intervention consistently demonstrate significant weight regain [38–40]. In our weight cycling animal model, we saw that rechallenging C57BL/6 J with WD feeding after weight loss resulted in rapid weight and fat mass regain, and recurrence of metabolic defects such as hyperinsulinemia. These defects were as severe as in the AOb mice and in some cases worse. Specifically, basal glucose uptake in eWAT was significantly lower and fasting insulin secretion significantly higher in WC animals compared to AOb and LOb at the 24-week time point. These findings suggest that a period of weight loss does not confer any significant long term benefits following weight regain, and in fact may cause more harm despite shorter overall exposure to WD. Future studies could utilise a LOb group fed WD for the same total duration as the WC group (16 wks) to further explore how the duration of WD exposure adversely impacts metabolism. Interestingly, although insulin secretion was not directly measured in DOz mice, the WC DOz mice did exhibit significantly higher HOMA-B scores at the 24 week timepoint when compared to LOb, indicating higher basal insulin secretion.

Next, we explored the cause of hyperinsulinemia in the WC C57BL/6 J. There were no significant differences in insulin-stimulated glucose uptake into adipose tissue nor any significant changes in hepatic triglyceride content, suggesting that hyperinsulinemia was not driven by increased insulin resistance in adipose tissue or liver. Ex-vivo islets from WC animals demonstrated hypersecretion of insulin in response to low but not high doses of glucose, suggesting that hyperinsulinemia was likely caused by elevated basal insulin secretion. Circulating FFA and FABP levels were not significantly higher in WC groups, suggesting hyperinsulinemia and hypersecretion of insulin are mediated through alternate pathways. Circulating metabolites including amino acids, lipid species, gut secreted hormones and other factors are known to modulate islet insulin secretion [41, 42]. Weight cycling therefore may result in an alteration in levels of such known or novel factors leading to a difference in insulin levels and islet insulin secretion between WC and LOb animals. Future studies could look to incubate isolated islets with plasma from WC mice to see if a circulating factor is responsible for differences in islet insulin secretion. Furthermore, plasma metabolomics and proteomics may help identify novel factors responsible for hyperinsulinemia in weight cycling animals.

The WC DOz mice were found to have lower fasting glucose, and higher HOMA-B scores than LOb controls. This may suggest a leftward shift in glucose stimulated insulin secretion (GSIS) perhaps via changes in glucokinase activity or the G6pc2/glucokinase futile cycle in pancreatic beta cells which influence the sensitivity of GSIS to glucose and regulate glycemia [43]. Increased ex-vivo islet insulin secretion at low but not high dose glucose in the WC C57BL/6 J mice similarly may indicate a leftward shift in GSIS. One limitation to this idea is that the low dose glucose of 2.8 mM used in the ex-vivo insulin secretion assays is far lower than the average in-vivo fasting glucose of 10.9 mM, restricting our ability to conclude that a leftward shift in GSIS truly exists in-vivo. A recent study found that ex-vivo islet insulin secretion was comparable between WC C57BL/6 J mice and controls with obesity, whereas arterial insulin AUC during a hyperglycemic clamp was significantly reduced, suggesting that a leftward shift in GSIS does not exist in-vivo at least in C57BL/6 J mice [28]. Winn et al. [28] additionally found that beta cell proliferation and expansion were stunted in WC mice compared to controls with obesity and transcripts of several key drivers of beta cell function were downregulated. Altogether, despite strain differences and some conflicting results, there is evidence both here and elsewhere indicating that weight cycling alters insulin secretory function of pancreatic beta cells.

In addition to a leftward shift in GSIS, another possibility is that the lower fasting glucose observed in the WC DOz mice is reflective of greater insulin sensitivity in the liver and direct suppression of hepatic glucose production [44]. However, weight cycling has previously been suggested to upregulate hepatic glucose production at least in C57BL/6 J mice [45]. In contrast to the results presented here, Kim et al. [45] additionally found fasting blood glucose to be elevated in the WC mice compared to controls, although these opposing results may stem from differences in the weight cycling protocols used.

A limitation of our study is that ex-vivo experiments were performed only in C57BL/6 J mice and extending such analysis into more diverse mouse strains is recommended. Recent documentation has demonstrated diversity in the changes in both insulin sensitivity and islet insulin following western diet feeding across mouse strains [18, 24]. Hence, given that DOz mice demonstrate tremendous variability in response to dietary change, exploration of tissue insulin sensitivity and islet insulin secretion in these mice may shed light on mechanisms driving the variable response. An additional limitation of the present study was that it only included male mice. Female mice are protected against many of the metabolic detriments of obesity and hence future studies should investigate potential sex effects on weight cycling [46]. Future studies could determine whether metabolic function is restored in WC DOz mice at the 16 week timepoint if mice are calorie restricted such that their body weight converges with that of the LOb group. Lastly, future weight cycling studies in DOz mice could increase sample size permitting genetic mapping of the diverse phenotypes present [17].

## Conclusion

Dietary intervention in C57BL/6 J mice results in rapid weight loss and is associated with improvement in metabolic complications and peripheral tissue insulin sensitivity. However, genetics greatly influences this response, as highlighted by both the differences observed between C57BL/6 J and DOz mice as well as the tremendous interindividual variability recorded in the genetically diverse DOz mice. Subsequent re-exposure to a Western diet results in rapid weight regain and recurrence of metabolic disturbances. Weight cycling animals overall are not protected from metabolic complications of obesity compared to always having obesity animals and develop more extreme hyperinsulinemia and hypersecretion of insulin compared to age-matched controls.

### Supplementary information

Supplemental Figure legends

Supplemental Figure 1

Supplemental Figure 2

## Data Availability

The data used in the present study are available upon reasonable request.
