# Supplementary material for: The metabolic consequences of ‘yo-yo’ dieting are markedly influenced by genetic diversity
Source: Int J Obes (Lond). 2024 Jul 3;48(8):1170–9. doi: 10.1038/s41366-024-01542-2 (PMC11281900; doi:10.1038/s41366-024-01542-2)
Supplement: Supplementary file 1 — Supplemental Figure legends [file 41366_2024_1542_MOESM1_ESM.docx]

**Supp. figure 1. Effect of Western-to-chow diet switch on metabolic outcomes in C57BL/6J mice**. a) Weight loss study design, b) body weight group means over 16 wks and at the 16 wk time point per group, c) liver triglycerides group means at the 16 wk time point per group, and d) Liver cholesterol group means at the 16 wk time point per group. Error bars are SEM. *p<0.05, **p<0.01, ***p<0.001 vs AOb (n=6-16/group). All statistical analysis was conducted using ANOVA. WL: Weight loss, AOb: Always having Obesity.

**Supp. figure 2. Effect of weight cycling on hepatic lipids in C57BL/6J mice.** a) Weight cycling experimental design for 24-wk study duration, group means at 24 wk time point for b) liver triglycerides and c) liver cholesterol. Error bars are SEM. *p<0.05, **p<0.01, ***p<0.001 vs chow (n=14-16/group). All statistical analysis was conducted using ANOVA. LOb: Later onset obesity, WC: Weight loss, AOb: Always having Obesity.
