# Supplementary figures and images for: The metabolic consequences of ‘yo-yo’ dieting are markedly influenced by genetic diversity
Source: Int J Obes (Lond). 2024 Jul 3;48(8):1170–9. doi: 10.1038/s41366-024-01542-2 (PMC11281900; doi:10.1038/s41366-024-01542-2)

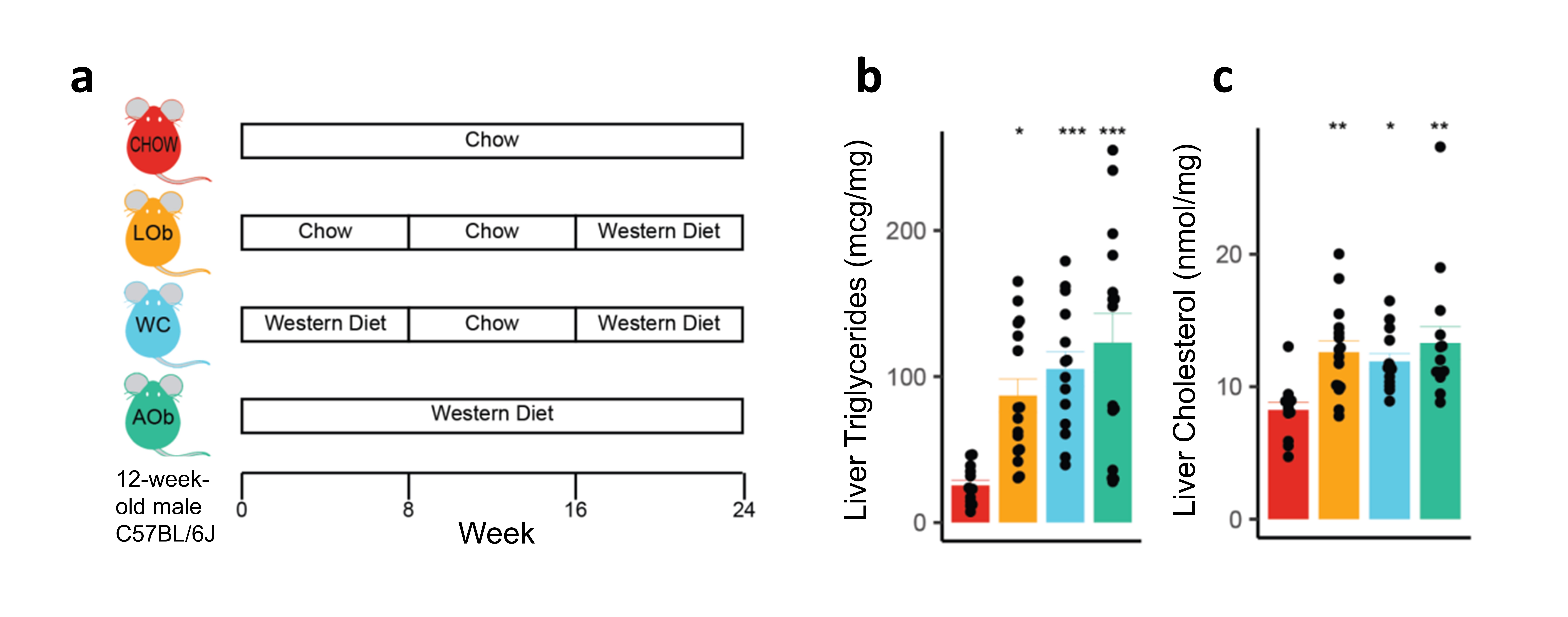

Supplement: Supplementary file 3 — Supplemental Figure 2 [file 41366_2024_1542_MOESM3_ESM.jpg]
